# Osteocalcin, Vascular Calcification, and Atherosclerosis: A Systematic Review and Meta-analysis

**DOI:** 10.3389/fendo.2017.00183

**Published:** 2017-07-31

**Authors:** Sophie A. Millar, Hinal Patel, Susan I. Anderson, Timothy J. England, Saoirse E. O’Sullivan

**Affiliations:** ^1^Division of Medical Sciences and Graduate Entry Medicine, School of Medicine, University of Nottingham, Royal Derby Hospital, Derby, United Kingdom

**Keywords:** osteocalcin, calcification, atherosclerosis, bone hormone, vascular disease, bone glutamic acid protein

## Abstract

**Background:**

Osteocalcin (OC) is an intriguing hormone, concomitantly being the most abundant non-collagenous peptide found in the mineralized matrix of bone, and expanding the endocrine function of the skeleton with far-reaching extra-osseous effects. A new line of enquiry between OC and vascular calcification has emerged in response to observations that the mechanism of vascular calcification resembles that of bone mineralisation. To date, studies have reported mixed results. This systematic review and meta-analysis aimed to identify any association between OC and vascular calcification and atherosclerosis.

**Methods and results:**

Databases were searched for original, peer reviewed human studies. A total of 1,453 articles were retrieved, of which 46 met the eligibility criteria. Overall 26 positive, 17 negative, and 29 neutral relationships were reported for assessments between OC (either concentration in blood, presence of OC-positive cells, or histological staining for OC) and extent of calcification or atherosclerosis. Studies that measured OC-positive cells or histological staining for OC reported positive relationships (11 studies). A higher percentage of Asian studies found a negative relationship (36%) in contrast to European studies (6%). Studies examining carboxylated and undercarboxylated forms of OC in the blood failed to report consistent results. The meta-analysis found no significant difference between OC concentration in the blood between patients with “atherosclerosis” and control (*p* = 0.13, *n* = 1,197).

**Conclusion:**

No definitive association was determined between OC and vascular calcification or atherosclerosis; however, the presence of OC-positive cells and histological staining had a consistent positive correlation with calcification or atherosclerosis. The review highlighted several themes, which may influence OC within differing populations leading to inconclusive results. Large, longitudinal studies are required to further current understanding of the clinical relevance of OC in vascular calcification and atherosclerosis.

## Introduction

Vascular calcification is a known major risk factor for mortality and morbidity and is an independent risk factor for cardiovascular disease (1–3). Vascular calcification, long believed to be a passive part of aging and “wear and tear,” is now considered an active, cell-mediated complex process that is regulated but not yet fully understood. Osteocalcin (OC) [also known as bone glutamic acid protein (BGLAP)] is an intriguing hormone produced by osteoblasts in bone that has been recently linked with an increasing number of extra-osseous biological roles and effects (4–8). One candidate thread of enquiry is its interaction with the vascular system, and its putative role in the process of vascular calcification or atherosclerosis. OC is not only produced by bone but is expressed by vascular smooth muscle cells (VSMCs) displaying an osteoblast-like phenotype (9).

Osteocalcin is the most abundant, non-collagenous component in the mineralized matrix of bone (10). The presence of three glutamic acid (Gla) residues allows for posttranslational γ-carboxylation at positions 17, 21, and 24. Between 60 and 90% of carboxylated OC (cOC) is deposited in the bone matrix; however, it can also be released into the circulation (11). OC can be undercarboxylated (ucOC) to differing degrees (from 0 to 2 carboxyl groups) due to decarboxylation, low activity of the vitamin K-dependent carboxylase enzyme, or vitamin K deficiency. ucOC has less affinity to hydroxyapatite and is more readily released into the circulation than cOC (12, 13).

ucOC has recently been appointed a predictor and potential therapeutic target of a number of diseases including diabetes and is believed to be the active form of OC (14). Studies have shown ucOC to be a regulator of pancreatic β cell and adipocyte gene expression, glucose metabolism and to increase insulin sensitivity in humans (14). Structural inconsistencies between ucOC and cOC have been explored, but discrepancies in reports are numerous and it is unknown the extent to which structural differences may play in their biological functions (15). It is hypothesized that ucOC may be the active form of OC involved in vascular calcification, but this has yet to be investigated.

Having established roles of other Gla containing proteins, such as Matrix Gla protein, in vascular calcification, many researchers have begun to explore the role of OC. Idelevich et al. investigated the effects of OC overexpressing mice cell lines (chondrocytes and VSMCs) (16). They showed that OC stimulates VSMC mineralization and differentiation, in particular through HIF-1α activation, surmising that OC fuels glucose utilization in VSMCs and promotes osteochondrogenic differentiation resulting in calcification. However, further studies are greatly lacking.

The aim of this systematic review and meta-analysis was to investigate and critically appraise the available literature linking OC to calcification and atherosclerosis in humans.

## Methods

### Search Strategy

The systematic review was carried out in accordance with the Meta-analysis Of Observational Studies in Epidemiology group proposal for reporting (17). A systematic and comprehensive search of PubMed and EMBASE (including Medline) was conducted to extract all articles examining an association between OC and vascular calcification or atherosclerosis. Identical search terms were used for both databases and included: “Osteocalcin AND Vascular Calcification,” “Osteocalcin AND Atherosclerosis,” “Osteocalcin AND Arterial Stiffness,” “Bone Gla Protein AND Vascular Calcification,” “Bone Gla Protein AND Atherosclerosis,” “Bone Gla Protein AND Arterial Stiffness,” “BGLAP AND Vascular Calcification,” “BGLAP AND Atherosclerosis,” “BGLAP AND Arterial Stiffness,” “Bone gamma-carboxyglutamic acid protein AND Vascular Calcification,” “Bone gamma-carboxyglutamic acid protein AND Atherosclerosis,” “Bone gamma-carboxyglutamic acid protein AND Arterial Stiffness,” “BGP AND Vascular Calcification,” “BGP AND Atherosclerosis,” “BGP AND Arterial Stiffness.” The searches were limited to include only human studies; with no restrictions on publication year, language, population or article type. Articles were subsequently excluded if the full text could not be found in English (*n* = 4). The searches were carried out by the 25/05/2017 with no year restrictions.

### Eligibility Criteria

The titles and abstracts for returned items were examined, and inappropriate articles were rejected. The criteria for inclusion was such that the article was an original, peer reviewed paper involving either longitudinal or cross-sectional human studies that investigated the relationship between OC and calcification or atherosclerosis. A further requirement of each study was that a form of OC must have been measured within their sample population. An endpoint relating specifically to the degree or severity of calcification or atherosclerosis was required to have been measured and reported. Studies that used assumptions of an increased risk of cardiovascular disease (CVD), e.g., by age and weight within their sample populations were excluded. All searches were conducted independently by two reviewers and compared. Where differing opinions on study eligibility existed (*n* = 6), they were discussed with the study principle investigator.

### Data Extraction and Analysis

The included articles were analyzed, and data were collated using an extraction form. The extracted data included the following: the population characteristics (age, sample size, ethnicity, and health status); type and method of OC measured; endpoint measurements; results of outcome and exposure measures; and the overall conclusions of the article and any key limitations or bias. A risk of bias assessment was performed according to the Cochrane Collaboration’s tool for assessing risk of bias (18).

### Statistical Analysis

A meta-analysis was performed on those studies that provided OC concentration in blood samples from an “increased vascular calcification/atherosclerosis group” and from a “control/healthy group.” Data were analyzed as forest plots using the Cochrane Review Manager software (Version 5.3. Copenhagen: The Nordic Cochrane Centre, The Cochrane Collaboration, 2014) and as funnel plots using Stata (StataCorp., 2009. Stat Statistical Software: Release 11. College Station, TX, USA). Funnel plot asymmetry (publication bias) was tested by Egger’s test (19). Since heterogeneity was expected between study protocols (different population characteristics, different methods of defining calcification or atherosclerosis, different specificity of assessment methods of OC) random-effect models were used. The results of continuous data of OC concentration are expressed as mean and SD. Where studies did not provide mean and SD, authors were contacted for data. In cases where no response from authors was obtained (*n* = 8), they could not be included in the statistical analysis (20–27). One author declined to supply requested information (28). Studies were weighted by sample size, and statistical significance was set at *p* < 0.05. The results are expressed as mean difference as all studies reported OC concentrations in the same units. It was not possible to perform further analyses due to large heterogeneity between studies.

## Results

**Figure 1:**
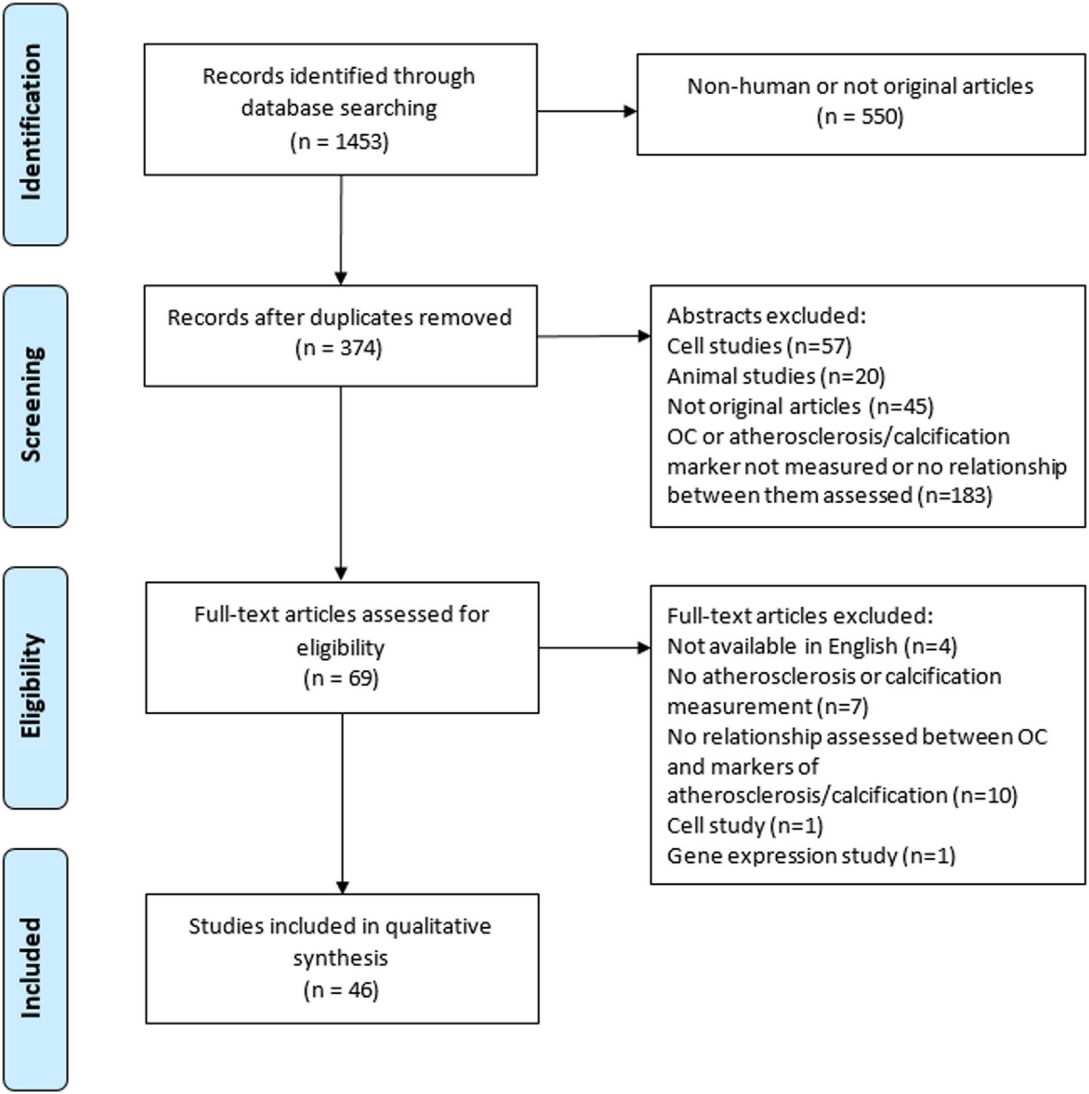
Flow chart for study retrieval and selection. Abbreviation: OC, osteocalcin.

The initial search yielded 1,453 records from which 374 abstracts were reviewed and 46 articles met the inclusion criteria (Figure 1). A description of each study is provided in Table 1. Of the 46 studies included in this review, 26 (56%) were designed specifically to examine the relationship between OC and markers of calcification or atherosclerosis (20, 22, 24–27, 29–47). The other 20 studies evaluated OC among a number of measurements, as a covariate, or in secondary analyses (21, 23, 28, 48–61). Forty-four out of the 46 studies were cross-sectional in design. Twenty-four studies did not make adjustments for any potential confounding variables, and 22 conducted multivariate analyses (Table 1). Ten studies had a sample size greater than 300 (20, 24, 25, 27, 30, 33, 35, 43, 45, 62).

**Table 1:** Summary of included studies.

Reference	*n*	Sex	Location	Inclusion criteria	OC measurement	OC type	Calcification or atherosclerosis measurement	Study outcomes	Adjustments
Levy et al. (46)	38	M, F	USA	Autopsy samples	RIA	Total	CS	OC was present in all calcified aortic tissue and heart valves and was either not detectable or present at very low levels in non-mineralized lesions and normal tissue	None
Jie et al. (36)	256	F	Netherlands	>55 years old	RIA	Total, free, and bound	CS	None	Age
Watson et al. (49)	173	M, F	USA	High and moderate risk for CHD	RIA	Total	CS	None	None
Bini et al. (57)	22	Not specified	USA	Human carotid endarterectomy specimens	Immunostaining	Total	CS	OC positively associated with calcification, progressing from type V to type VI lesions	None
Montalcini et al. (50)	157	F	Italy	Postmenopausal women aged 45–75 years	RIA	Total	C-IMT	Positive relationship between OC and carotid atherosclerosis prevalence in those with low BMD	Age, systemic hypertension, hyperlipidemia, DM, obesity, smoking
Iba et al. (56)	135	F	Japan	Postmenopausal osteoporotic women	ELISA	Total	ACS	None	None
Rajamannan et al. (59)	58	M, F	USA	T2DM, >18 years old, cardiac valve surgery	Immunostaining	Total	CS	OC expression was upregulated in the calcified rheumatic valves and was present at low levels in the degenerative mitral valves	None
Göossl et al. (37)	72	M, F	USA	Coronary atherosclerosis patients	Flow cytometry	OCN+ EPCs	CA and endothelial function	Positive relationship between OC+ cells and stage of coronary atherosclerosis	None
Kanazawa et al. (35)	328	M, F	Japan	T2DM	RIA	Total	PWV and C-IMT	Negative correlation between OC and PWV and C-IMT in men only	None
Pal et al. (40)	23	M, F	Australia	Peripheral artery disease	Flow cytometry	OC + MNCs	ACS	Positive relationship between OC+ cells and aortic calcification	None
Foresta et al. (38)	35	M	Italy	Erectile dysfunction patients	Flow cytometry	OC + EPCs	C-IMT	Positive relationship between OC+ cells and IMT	None
Zhang et al. (25)	461	M, F	China	Chest pain, heaviness, periodic discomfort, and palpitations	ELISA	Total	CA	OC was significantly higher in those with 0 diseased vessel than in those with 1,2, or ≥3 diseased vessels	None
Parker et al. (27)	363	F	USA	≥65 years old	ELISA	Total	ACS	None	Age, CVD risk factors, BMD, mineral metabolism, estrogen use, kidney function, vitamin D, PTH, and BSAP
Okura et al. (31)	92	M, F	Japan	Essential hypertension	ELISA	ucOC	C-IMT	Positive relationship between ucOC levels and calcification	None
Awan et al. (55)	19	M, F	Canada	Familial hypercholesterolemia	ELISA	Total	CACS	Negative correlations between ACS and OC	None
Bao et al. (26)	181	M	China	MS, CA	ELISA	Total	CA	Negative relationship between OC and number of stenotic vessels in subgroup analysis with NGTnormal glucose tolerance (*n* = 60)	None
Pirro et al. (61)	120	F	Italy	Newly diagnosed, never-treated postmenopausal osteoporosis	FACS	OCN+ OPCs	PWV	Moderate positive correlation between AoPWV and OC+ cells	Age, smoking status, waist circumference, SBP (or alternatively MABP), heart rate, glucose, cholesterol, TG, PTH, osteoporotic status, and the log-transformed count of CD34+/AP+ cells
Kanazawa et al. (47)	50	M, F	Japan	T2DM	RIA	Total	C-IMT	Positive correlation of baseline plaque score with changes in OC,^a^ and negative correlation of changes in OC with changes in plaque score^b^	^a^^,^^b^
Reyes-Garcia et al. (34)	78	M, F	Spain	T2DM	RIA	Total	C-IMT	Positive association between OC and IMT, carotid plaques, and aortic calcifications, in women only	None
Kim et al. (30)	769	F	Korea	Women	ELISA	Total	ACS	Negative relationship between OC and ACS	Age
Ogawa-FuruyaOgawa et al. (29)	218	M, F	Japan	T2DM	RIA	Total, ucOC	ACS	Negative association between both serum OC and ucOC concentrations and an ACS of 3 and greater, in men only	Age, BMI, serum creatinine and LDL-c, radial BMD, smoking, duration of DM, HbA1c, and HOMA-IR
Janda et al. (48)	67	M, F	Poland	>18 years old, PD ≥2 months, negative history of neoplastic diseases	ELISA	Total	C-IMT	OC positively associated with C-IMT	Age and major CVD risk factors
Sheng et al. (33)	817	M, F	China	>50 years old, T2DM	RIA	Total	C-IMT and plaques	Negative association between OC, carotid plaques,^c^ and C-IMT^d^	^c^^,^^d^
Janda et al. (23)	59	M, F	Poland	ESRD	ELISA	Total	CS	None	Low HDL-c (<1.0 mmol/L in men, <1.3 mmol/L in women), high TG (>1.7 mmol/L), and high BMI (≥25 kg/m^2^), as well as hypertension, CRP, gender, dialysis status of patients, and Ca × Pi
Foresta et al. (39)	3	M	Italy	Carotid endarterectomy	Immunostaining	Total	CS	Positive relationship observed between OC and extent of calcification in lesions/necrotic core (no OC detected in corresponding healthy portions of carotid wall specimens)	None
Yang et al. (20)	1,319	F	China	Postmenopausal women	ELISA	Total	C-IMT	Negative correlation between OC and C-IMT	Age, years since menopause, BMI, waist circumference, SBP, DBP, homeostasis model assessment, insulin resistance, TG, HDL-c, CRP, smoking, antidiabetic therapy, antihypertensive therapy, lipid lowering therapy, and family history of CVD
O’Neill and Adams (58)	19	F	USA	Mastectomy, partial mastectomy, or lumpectomy patients and a diagnosis of ESRD or CKD	Immunostaining	Total	CS	OC positively related with more heavily calcified arteries and appeared to coincide with calcium deposits	None
Ishimura et al. (28)	167	M	Japan	Stable HD for >3 months	RIA	Total	CS	None	None
Dweck et al. (54)	30	M, F	UK	Valve replacement surgery or asymptomatic disease under surveillance	Immunostaining	Total	18F-NaF	Positive relationship between aortic valve 18F-NaF uptake and OC	None
Krzanowski et al. (51)	57	M, F	Poland	>18 years old, stable dialysis ≥2 months, negative history of malignant disease, and lack of active viral infection	ELISA	Total	PWV	Negative relationship between OC and PWV	Age, hypertension, MABP, AoPWV evaluation, hypertension, ln (dialysis therapy duration), dialysis fluid exchange method, and Ca Pi index
Ma et al. (24)	1,077	M	China	Males with and without NGT	ELISA	Total	C-IMT and plaques	Negative relationship between OC and carotid plaque in subgroup analysis with men with NGT (*n* = 638). No associations with C-IMT	Age, BMI, WHR, FBG, PPG, SBP, DBP, TG, HDL-c, LDL-c, smoking, logHOMA-IR, and logHOMA-%B
Prats-Puig et al. (32)	203	M, F	Spain	5–10 years old, MS families, and no pubertal development	ELISA	Total, ucOC	C-IMT	ucOC positively associated with C-IMT in MS+ family offspring. Total OC not associated	Age, gender, BMI, fat mass, HOMA-IR, serum lipids, and CRP
Choi et al. (41)	162	M, F	Korea	Healthy adults	ECL and ELISA	OC, ucOC	CACS	Positive relationship between OC and CACS in men only. No significant findings for ucOC	Age, BMI, smoking, hypertension, diabetes, SBP, HOMA2-IR (log-transformed), TG (log-transformed), HDL-c, and lumbar BMD
Zhang et al. (42)	224	M, F	China	CA	Flow cytometry	OC + EPCs	CACS	No correlation between OC+ cells with calcification in stable angina pectoris patients. In unstable angina pectoris and acute myocardial infarction patients, the number of spotty calcium deposits was significantly positively correlated with the absolute numbers of OC+ cells	None
Maser et al. (60)	50	M	USA	>18 years old, T2DM	ELISA	Total, ucOC	CACS	None	Age, duration of diabetes, HOMA-IR, BMI, gender, SBP, HbA1c, leptin, and adiponectin
Collin et al. (52)	23	M, F	USA	18–85 years old, early atherosclerosis	Flow cytometry	OC + MNCs	CA, endothelium dependent coronary vasoreactivity	Positive relationship between OC+ cells and extent of necrotic core and calcification	None
Luo et al. (45)	476	M, F	China	BMI ≥18.5 and <25.0 kg/m^2^, NGT, normotensive, and normal lipid status	ELISA	Total	C-IMT	None	Age, BMI, W, SBP, DBP, FPG, serum fasting insulin, CRP, smoking status, and CVD family history
Zhang et al. (22)	290	M, F	China	Non-dialysis CKD patients	ELISA	ucOC	C-IMT	Negative relationship between ucOC and carotid plaques^e^ and IMT^f^	^e^^,^^f^
Janda et al. (21)	59	M, F	Poland	HD and PD patients	ELISA	Total	C-IMT	No significant correlations	FBG, PTX3, FRS, and dialysis status
Yang et al. (53)	421	M, F	China	CA and echocardiography	ELISA	Total	Echocardiography	Positive relationship between OC and aortic valve stenosis	None
Ramirez-Sandoval et al. (63)	76	M, F	Mexico	PD patients ≥6 months; stable clinical course ≥3 months	Luminometry	Total	CS	None	None
Golovkin et al. (64)	112	M	Russia	CAD patients	ELISA	Total	CS	Levels of OC higher in patients with mild CS than those with severe calcification when assessed by Agatston score, but not Syntax score	None
Barbarash et al. (65)	112	M	Russia	Age ≤75 years; diagnosis of stable angina according to the Canadian Cardiovascular Society guidelines	ELISA	Total	CS	None	None
Yun et al. (43)	3,604	M, F	Korea	Healthy adults	ECL	Total	PWV	OC level independently related to arterial stiffness; inverse J shape relationship. At low OC levels, the relationship was negatively linear. However, after controls for age and metabolic factors, the relationship with arterial stiffness at high levels of OC was not significant	Age, BMI, SBP, glucose, TV, eGFR, smoking, drinking, exercise, menopause, history of hypertension and diabetes, and total hip BMD
Kim et al. (44)	122	M	Korea	CABG	ELISA	cOC, ucOC	CACS	No significant differences in ucOCN or cOCN levels between groups divided according to CAC score	Age, BMI, T2DM status, hypertension, SBP, DBP, HbA1c, TC, creatinine, and statin therapy
Yang et al. (62)	593	M, F	USA	Patients undergoing CA because of known or suspected CAD	Flow cytometry	Total	CAS	OC+ early EPCs associated with an increase in levels^g^ and risk^h^ of higher degree of severity of CAS	^g^^,^^h^

*M, male; F, female; T2DM, type 2 diabetes mellitus; RIA, radioimmunoassay; ucOC, undercarboxylated osteocalcin; ACS, aortic calcification score; ELISA, enzyme-linked immunosorbent assay; C-IMT, carotid intima-media thickness; MS, metabolic syndrome; PD, peritoneal dialysis; CA, coronary angiography; PWV, pulse wave velocity; CS, calcification score; OC, osteocalcin; EPC, endothelial progenitor cells; FACS, fluorescence activated cell sorting; MNCs, mononuclear cells; cOC, carboxylated osteocalcin; CACS, coronary artery calcification score; ECL, electrogenerated chemiluminescence; CABG, coronary artery bypass grafting; BMI, body mass index; CKD, chronic kidney disease; HD, hemodialysis; ESRD, end-stage renal disease; OPCs, osteoprogenitor cells; LDL-c, low density lipoprotein cholesterol; BMD, bone mineral density; HOMA-IR, homeostasis model assessment index for insulin resistance; CVD, cardiovascular disease; PTH, parathyroid hormone; BSAP, bone-specific alkaline phosphatase; CRP, C-reactive protein; HDL, high-density lipoprotein; CHD, coronary heart disease; NGT, normal glucose tolerance; WHR, waist-to-hip ratio; FBG, fasting blood glucose; PPG, oral glucose challenge; SBP, systolic blood pressure; DBP, diastolic blood pressure; TG, triglycerides; HOMA-%B, beta cell function; eGFR, estimated glomerular filtration rate; W, weight; MABP, mean arterial blood pressure; TC, total cholesterol; PTX3, pentraxin 3; FRS, Framingham Risk Score; CAD, coronary artery disease; CAS, coronary artery stenosis*.

*^a^Duration of diabetes and Brinkman index*.

*^b^Age, duration of diabetes, gender, BMI, Brinkman index, SBP, serum creatinine, LDL-c, HDL-c, TG, and HbA1c*.

*^c^Age, gender, smoking, alcohol intake, duration of diabetes, BMI, waist circumference, SBP, DBP, HOMA-IR, CRP, FBG, serum creatinine, serum urea, serum cholesterol, TG, HDL-c, and LDL-c*.

*^d^Age, gender HbA1c, HOMA-IR, and serum CRP*.

*^e^Age, sex, BMI, smoking history, MABP, eGFR, therapeutic medication use, FBG, TC, TG, LDL-c, HDL-c, and hs-CRP levels*.

*^f^Age*.

*^g^None*.

*^h^Age, sex, hypertension, diabetes, hypercholesterolemia, smoking, obesity, and family history of premature CAD*.

Thirty-three studies (72%) measured OC by enzyme-linked immunosorbent assay, electrogenerated chemiluminescence, or radioimmunoassay, while the remaining studies used flow cytometry or fluorescence activated cell sorting methods, or examined OC by histological immunostaining (Table 1). OC was measured using luminometry in one study (63). ucOC was measured in five studies (22, 31, 32, 41, 44), cOC in one study (44), and total OC was measured in the remaining studies (Table 1). OC positive mononuclear cells, endothelial progenitor cells (EPCs), or osteoprogenitor cells (OPCs) were examined by seven studies (37, 38, 40, 42, 52, 61, 62).

Methods of calcification or atherosclerosis measurements used in OC analyses varied and ranged from calcification scoring methods (*n* = 22), intima-media thickness measurements (*n* = 14), pulse wave velocity (PWV) measurements (*n* = 4), plaque presence (*n* = 2), and coronary angiography or echocardiography (*n* = 6; Table 1). One study used 18F-Sodium Fluoride uptake as a marker of calcification (54).

### Risk of Bias Assessment

**Figure 2:**
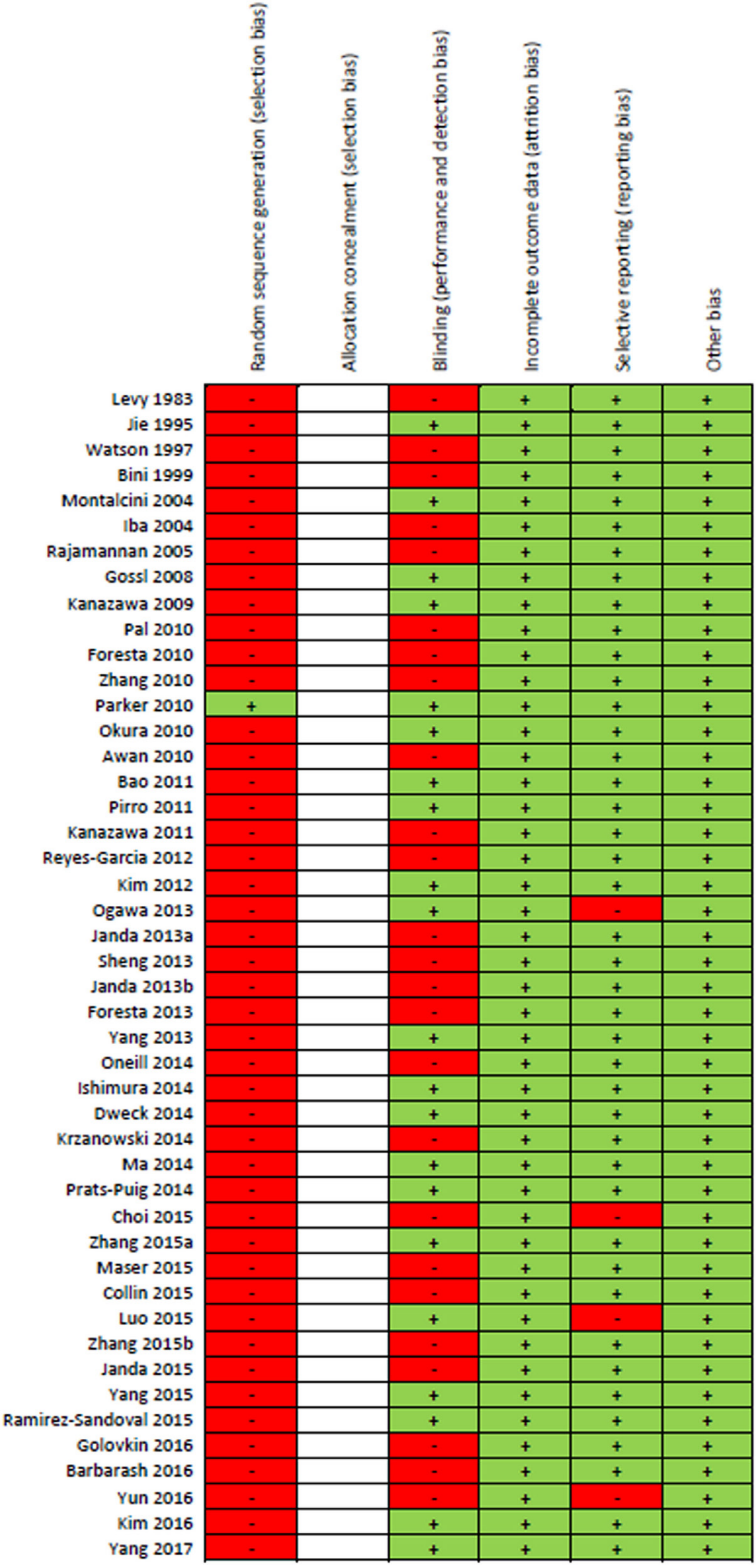
“Risk of bias” summary: green (+) indicates low-bias risk and red (–) indicates high-bias risk. The studies included in this review were all observational in study design and thus the risk of bias for the item “allocation concealment” was not performed and spaces were left blank.

Results of the risk of bias assessment for all 46 studies are presented in Figure 2. Due to the majority of studies being cross-sectional in design and limitations on sample sizes, only one study randomly selected participants. Since all the included studies were observational cohort studies, no risk of bias assessment for “Allocation concealment” could be performed. Forty-six percent of studies included a component of blinding. None of the studies were reported with high risk of attrition bias or other bias. Four studies were reported with high risk of reporting bias. Overall, most information was from studies at low risk of bias.

### Relationship between OC and Markers of Atherosclerosis or Calcification

**Figure 3:**
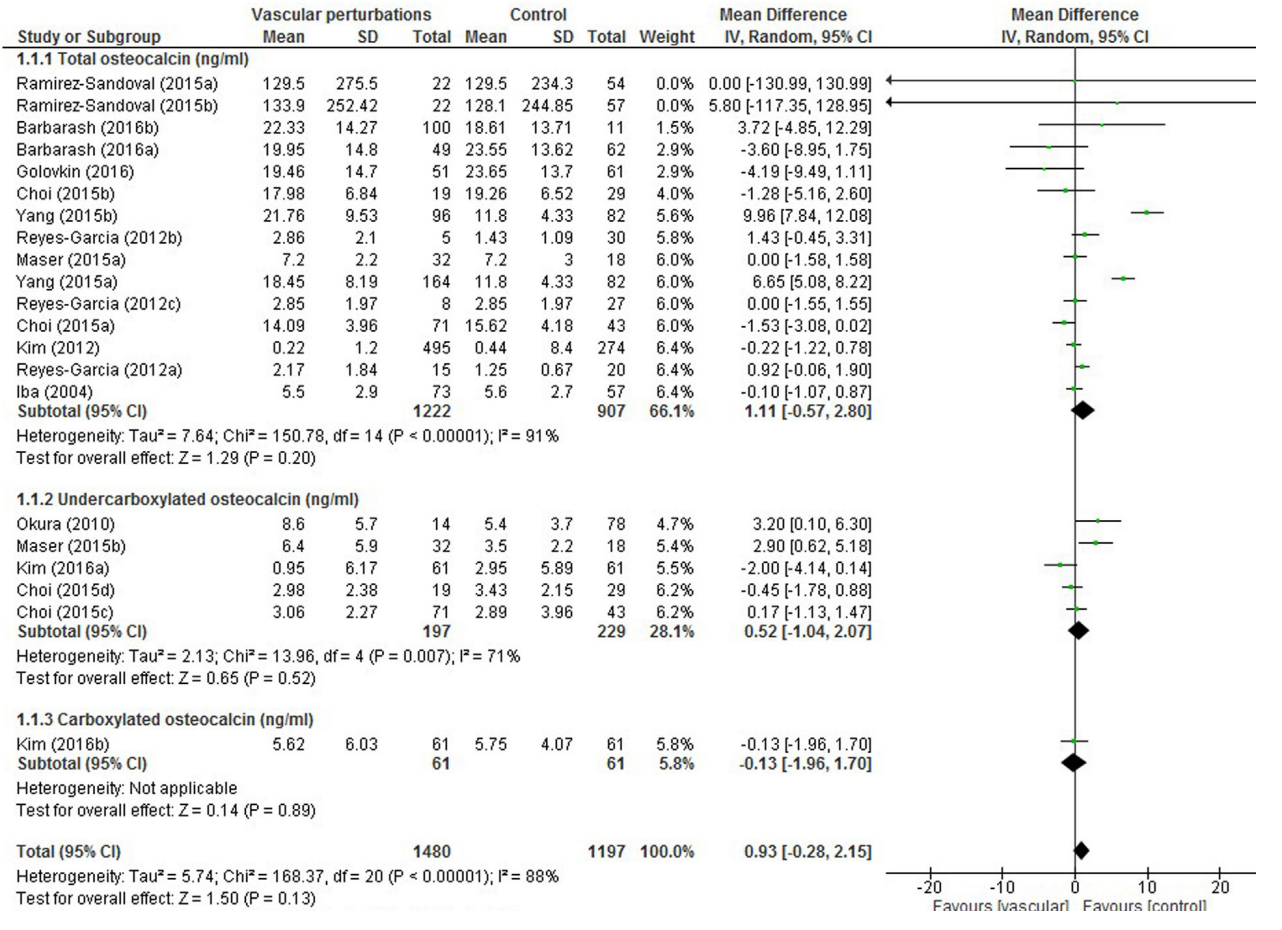
Meta-analysis examining osteocalcin (OC) concentration (nanograms per millilitre) differences between groups with and without vascular perturbations (markers of calcification or atherosclerosis).

**Figure 4:**
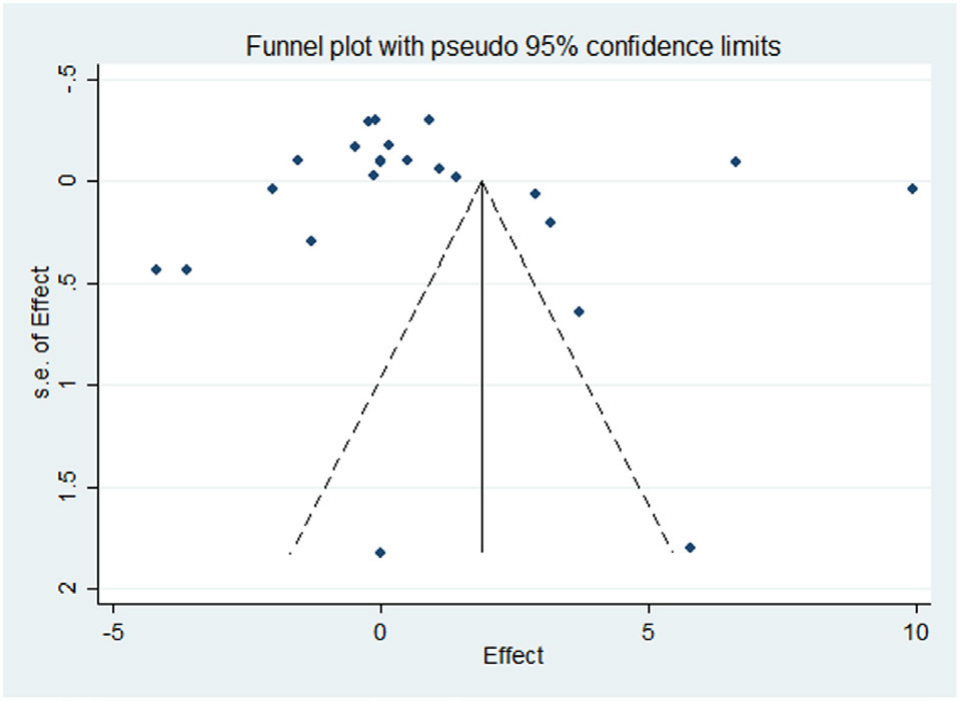
Funnel plot evaluating publication bias on the effect of OC concentration on atherosclerosis or calcification. The SE of the mean difference in osteocalcin concentration for each study is plotted against its effect size (horizontal axis). Although the distribution of the studies within the funnel plot does not appear symmetrical, there was no statistical evidence of publication bias (Egger’s statistic *p* = 0.279).

Results of the meta-analysis examining OC concentrations between groups with normal vascular parameters and those presenting with markers of calcification/atherosclerosis are detailed in Figure 3. There was no significant overall difference between OC concentration (total, ucOC, or cOC) in patients with “atherosclerosis” and control, though a trend toward lower OC concentrations was seen in the control group [overall mean difference 0.93 ng/mL (95% CI −0.28, 2.15), *p* = 0.13]. There was significant statistical heterogeneity, *I*^2^ 88%, *p* < 0.00001. Egger’s test showed no publication bias present (*p* = 0.279, Figure 4).

Due to multiple end points and assessments within studies, results are reported in terms of total outcomes to avoid bias reporting of overall positive, negative or neutral findings per study in the semiquantitative analysis. Among the studies, the relationship between OC and markers of atherosclerosis or calcification was reported as positive for 26 outcomes and negative for 17 outcomes, while no relationship was established for 29 outcomes.

No significant relationship was established for cOC, which was measured by only one study (44). In seven studies that measured ucOC and markers of atherosclerosis or calcification, two positive outcomes were reported (31, 32), three negative outcomes (22, 29), and four non-significant outcomes (41, 44, 60). Fifty-four percent of studies did not adjust for any confounding variables (Table 1). Of these, 17 positive outcomes between OC and markers of atherosclerosis or calcification were reported, 5 negative outcomes, and 12 non-significant outcomes. Within the other 22 studies adjusting for age and/or other confounding variables including CVD risk factors, 8 positive outcomes were reported between OC and markers of atherosclerosis or calcification, 11 negative outcomes were reported, and 17 outcomes were non-significant.

All 13 studies measuring OC positive mononuclear cells, EPCs, or OPCs, or histological staining for OC, reported a positive relationship between OC and markers of atherosclerosis.

A number of different outcomes were reported within the same studies, depending on gender, type of OC measured, or type of calcification or atherosclerosis measurement. Ogawa-Furuya et al. (29) and Kanazawa et al. (35) found a negative association between OC and markers of atherosclerosis within men but no significant association in women (29, 35). Prats-Puig et al. (32) found a positive association with ucOC, but no significant association was reported for total OC (32). Reyes-Garcia et al. (34) found a positive association in women only. Ma et al. (24) found an association between OC and plaque scores within men with normal glucose tolerance, but no association was reported with C-IMT (24). Choi et al. (41) found a positive association in men with total OC but no association in women or with ucOC (41). Yun et al. (43) found a negative association at low levels of OC only (43), and Zhang et al. (42) found an association in unstable angina pectoris and acute myocardial infarction patients but not in pectoris patients with stable angina (42). The single longitudinal study by Kanazawa et al. (47) showed conflicting results as baseline measurements demonstrated that OC was significantly and positively correlated with plaque score; however, OC was then negatively correlated with changes in plaque score at the end of the study (47).

Overall, in studies that conducted gender sub-analyses, more positive relationship outcomes between OC and measurements of atherosclerosis or calcification were observed within males than negative outcomes, while the reverse was reported within females. A neutral outcome was the most common finding overall for males and females. For the remaining studies when the study population was analyzed as a total, a positive outcome was the most common finding.

Thirty-six percent of outcomes from studies conducted in Asia found a negative relationship between OC and markers of atherosclerosis or calcification, in contrast to 6% in European studies and 5% in American, Canadian, Mexican, or Australian studies. Outcomes examined by population characteristics, e.g., chronic kidney disease patients, healthy adults, vascular problems (vascular dysfunction/coronary heart disease/atherosclerosis) were mixed. When examining studies that only used blood samples to measure OC (*n* = 33), i.e., excluding the OC positive cell studies and histological studies, no trend became apparent.

No trend was observed for differing methods of measuring calcification or atherosclerosis, and results were similar when examining total studies and those that used blood samples to assess OC. Overall, the method of calcification scoring to assess calcification or atherosclerosis resulted in the most non-significant outcomes with OC measurements.

## Discussion

This review aimed to uncover whether there was a conclusive association between OC and vascular calcification or atherosclerosis in humans by performing a systematic review of the current literature. In total, 33 studies measuring blood OC concentrations and 13 studies measuring OC positive cells or histological staining of OC were found through the literature searches. Overall, no clear association could be made between OC and extent of calcification or atherosclerosis, which was confirmed by meta-analysis. However, all studies measuring OC positive cells or histological staining of OC showed a positive relationship with calcification or atherosclerosis. Some potential reasons for discrepancies in results were explored during the synthesis, including the method of OC measurement, variability in population characteristics and ethnicity, gender, and method of measuring calcification or atherosclerosis.

The majority of studies measured blood concentrations of total OC, ucOC, or both, while only one study measured cOC. No significant relationship was established for cOC, and the studies measuring total OC and/or ucOC resulted in a combination of positively and negatively correlated associations, as well as non-significant outcomes. Within the current review, the role of the different forms of OC in the vasculature, i.e., ucOC or cOC, could not be determined as too few studies examined OC in its different presentations. Total OC may not be a valuable measurement for risk of vascular calcification as it is suggested that ucOC is the biologically active form. Future research may benefit from focusing on the various types of OC to ascertain whether there is a relationship present. However, there has been difficulty in measuring ucOC and cOC as few assays exist and it is unclear which assay system provides the most accurate measurements due to problems with comparability and heterogeneity of OC (14, 66–68). OC also displays a circadian rhythmicity with a nocturnal peak and thus timing of blood sampling may also contribute to variations in results (69, 70).

Circulating mononuclear cells, EPCs, and OPCs expressing OC were used by studies included in this review as a form of OC measurement. These cells are found in the bloodstream and released by bone marrow. EPCs can differentiate into endothelial cells and play a role in angiogenesis (71). It has been hypothesized that OC positive EPCs are involved in the mechanism of calcification by mediating abnormal vascular repair. This is thought to be as a result of the activation of osteogenic genes within the EPCs. EPCs are considered to be part of the initial response to vessel damage; however, instead of promoting normal repair they express an “osteogenic transcriptosome” which promotes calcification. This is further supported by gene expression analyses of CD34+ cells showing expression of bone mineralization related proteins such as Runx2 and BMP-2. It has been proven that OC is expressed by atherosclerotic plaques and VSMCs, which have differentiated as part of the process of calcification already (72) and so studying the association between cells expressing OC and calcification could provide further direction for future studies. All the articles reviewed in this study found a significant, positive correlation between OC positive cells and increased calcification or atherosclerosis.

Histological staining for OC resulted in similarly positive findings. The positive correlation found in all these studies between OC and calcification supports the hypothesis that OC expressed by these cells may contribute to the initial calcification of the vessels. This is comparable to Idelevich et al. (16) whose observations suggest OC is an active contributor to the mineralization process and stimulates differentiation of chondrocytes and VSMCs (16). These observations indicate the potential clinical implications of OC in detecting subclinical atherosclerosis and spotty calcifications. Conflicting results arise only when OC is measured in blood samples, suggesting a need for ucOC and cOC to be measured separately with reliable reproducible assays to disentangle their functions.

All the studies examined in this systematic review, except two, were cross-sectional observational studies. These, although useful, are also limited in their interpretation as a cause–effect relationship cannot be concluded from the results. The mechanism behind calcification remains very much unresolved and so the role, if any, of OC in the process is difficult to identify. The longitudinal study by Kanazawa et al. showed conflicting results between baseline measurements and final measurements. These results demonstrated that initially, total OC was significantly and positively correlated with carotid plaque score (47). However, OC was negatively correlated with changes in plaque score even after adjustment with atherosclerosis-related risk factors at the end of the study. This suggested that OC was relevant to calcification at both extremes, forming a U-shaped association. It was therefore hypothesized that atherosclerotic plaques may initially promote OC secretion but eventually the increased level of OC may suppress the progression of atherosclerosis or calcification. Furthermore, the longitudinal study by Yang et al. (62) reported that very high numbers of early circulating OC positive EPCs tended to be associated with to the risk of all-cause mortality (62). Further studies in a similar prospective longitudinal style should be carried out to confirm the hypotheses suggested. This may provide a reason for the numerous conflicting cross-sectional studies that have studied populations at different phases of disease.

No clear trends could be seen as a result of gender, although it can be noted more negative than positive outcomes between OC and calcification or atherosclerosis were observed in men, while the opposite was observed for women. Gender differences in OC actions have been reported elsewhere, for example in diabetes and fertility (5, 73). Due to the variety of population characteristics included the studies reviewed, associations could not be concluded between particular populations and study results. Most participants were over 50 years of age (data not shown), and this may seem reasonable as the risk of vascular calcification and atherosclerosis increases with age; however, with an increase in age also comes an increase in comorbidities, which could have influenced the results found in some studies. In addition, OC concentrations are influenced by medication including glucocorticoid therapy, antiresorptive agents and vitamin D treatment (74). Not all the studies accounted for these being potential confounding factors.

An interesting study by Namba et al. (75) examined the effect on bone metabolism markers and atherosclerosis measures in patients with atrial fibrillation when switching from warfarin (a vitamin K antagonist) to rivaroxaban (75). This study found ucOC concentrations to decrease after 6 months of rivaroxaban treatment as vitamin K was no longer prohibited. Concomitantly, osteopontin (an atherosclerosis-related marker) was decreased, bone alkaline phosphatase (a bone formation marker) was increased and PWV and augmentation index were significantly decreased. The availability of vitamin K allows for γ-carboxylation of ucOC to cOCN, and the reported improvements in atherosclerosis markers suggest and allude to the importance and potential clinical relevance of the differing presentations of OC, and their usefulness to detect at risk populations.

Ethnicity may play a role in the conflicting results of the studies in this review. Thirty-seven percent of studies conducted in Asia reported negative relationships between OC and calcification or atherosclerosis, compared to 6% of European studies. Studies in both populations used a combination of endpoints measuring calcification or atherosclerosis, showing that this variation did not affect this comparison. Within 10 studies that had a sample size >300, 1 reported a positive outcome, 7 reported negative outcomes, with the remaining two finding no significant outcomes. Eight of these larger studies were conducted in Asia, thus the negative outcomes may be reflective of higher statistical power or ethnicity or a combination of both.

The meta-analysis performed providing adequate data on OC concentration confirmed findings from the qualitative component of this systematic review. The large heterogeneity reported again questions the reliability of serum or plasma measurements of OC concentration, the accuracy of methods of measurement of total OC and its undercarboxylated and carboxylated forms, and the need for well-defined studies with a primary aim of assessing OC’s role in vascular calcification and atherosclerosis. The heterogeneity present in the meta-analysis can be further explained by the variety of study populations (kidney disease, diabetes or glucose intolerance, postmenopausal women, and hypertension) and the different methods employed to distinguish between those with and without vascular calcification or atherosclerosis and varying severities therein.

There are a few limitations in this review, which should be considered. Despite a thorough search of the two databases chosen, the addition of more databases may have widened the search to increase the number of results and hence improve the reliability and validity of the findings. However, the review was carried out by two independent reviewers, and searches generated were analyzed separately and then compared. Only one study analyzed cOC, which limits the results reported here. Furthermore, due to the observational nature of the studies included, only associations can be drawn and no causal relationships can be concluded.

In conclusion, no clear association can be made between OC and vascular calcification or atherosclerosis from the currently available published research. This review has highlighted themes, which may influence OC within differing populations leading to inconclusive results. In addition, the various forms of circulating OC should be separately measured and considered in future studies. Longitudinal studies may provide more insightful results as to the potential pathological effects of OC.

## Author Contributions

SM and SO: substantial contributions to the conception or design of the work. All the authors: the analysis and interpretation of data for the work; drafting the work or revising it critically for important intellectual content; final approval of the version to be published; and agreement to be accountable for all aspects of the work in ensuring that questions related to the accuracy or integrity of any part of the work are appropriately investigated and resolved.

## Conflict of Interest Statement

The authors declare that there is no conflict of interest that could be perceived as prejudicing the impartiality of the research reported and that there is no other financial or other potential conflict of interest. The reviewer, KH, and handling editor declared their shared affiliation, and the handling editor states that the process nevertheless met the standards of a fair and objective review.
